# Supplementary material for: Retrospective Multicentric Study on Non-Optic CNS Tumors in Children and Adolescents with Neurofibromatosis Type 1
Source: Cancers (Basel). 2020 May 31;12(6):1426. doi: 10.3390/cancers12061426 (PMC7353051; doi:10.3390/cancers12061426)
Supplement: Supplementary file 1 [file cancers-12-01426-s001.pdf]

cancers

MDPI

Article

Retrospective Multicentric Study on Non-Optic CNS Tumors in Children and Adolescents with Neurofibromatosis Type 1

Claudia Santoro, Stefania Picariello, Federica Palladino, Pietro Spennato, Daniela Melis, Jonathan Roth, Mario Cirillo, Lucia Quaglietta, Alessandra D'Amico, Giuseppina Gaudino, Maria Chiara Meucci, Ursula Ferrara, Shlomi Constantini, Silverio Perrotta and Giuseppe Cinalli

Supplementary Table S1. Univariate Cox regression analysis of progression free survival in patients with low grade gliomas.

	Hazard Ratio (95%CI)	*p*
**Multiple lesions**	2.81 (1.06–7.46)	**0.038**
**Optic pathway glioma co-presence**	1.24 (0.52–2.97)	0.629
**Surgery first approach**	0.48 (0.22–1.04)	0.062
**Age at diagnosis (continuous variable)**	0.99 (0.99–1.01)	0.902
**Age at diagnosis < 10 years**	1.12 (0.46–2.71)	0.803
**Female gender**	1.27 (0.81–2.00)	0.292
**Inherited NF1**	1.37 (0.86–2.19)	0.189
**Symptomatic at diagnosis**	0.46 (0.17–1.26)	0.131
**Brainstem vs other locations**	0.73 (0.28–1.90)	0.519
**Posterior fossa vs other locations**	1.07 (0.38–3.03)	0.895
